# Regenerative Approach to Bilateral Rostral Mandibular Reconstruction in a Case Series of Dogs

**DOI:** 10.3389/fvets.2015.00004

**Published:** 2015-03-30

**Authors:** Boaz Arzi, Derek D. Cissell, Rachel E. Pollard, Frank J. M. Verstraete

**Affiliations:** ^1^Department of Surgical and Radiological Sciences, School of Veterinary Medicine, University of California Davis, Davis, CA, USA; ^2^Department of Biomedical Engineering, University of California Davis, Davis, CA, USA

**Keywords:** mandible, reconstruction, bone morphogenetic proteins, 3D printing, regeneration, dog

## Abstract

Extensive rostral mandibulectomy in dogs typically results in instability of the mandibles that may lead to malocclusion, difficulty in prehension, mastication, and pain of the temporomandibular joint. Large rostral mandibular defects are challenging to reconstruct due to the complex geometry of this region. In order to restore mandibular continuity and stability following extensive rostral mandibulectomy, we developed a surgical technique using a combination of intraoral and extraoral approaches, a locking titanium plate, and a compression resistant matrix (CRM) infused with rhBMP-2. Furthermore, surgical planning that consisted of computed tomographic (CT) scanning and 3D model printing was utilized. We describe a regenerative surgical technique for immediate or delayed reconstruction of critical-size rostral mandibular defects in five dogs. Three dogs had healed with intact gingival covering over the mandibular defect and had immediate return to normal function and occlusion. Two dogs had the complication of focal plate exposure and dehiscence, which was corrected with mucosal flaps and suturing; these dogs have since healed with intact gingival covering over the mandibular defect. Mineralized tissue formation was palpated clinically within 2 weeks and solid bone formation within 3 months. CT findings at 6 months postoperatively demonstrated that the newly regenerated mandibular bone had increased in mineral volume with evidence of integration between the native bone, new bone, and CRM compared to the immediate postoperative CT. We conclude that rostral mandibular reconstruction using a regenerative approach provides an excellent solution for restoring mandibular continuity and preventing mandibular instability in dogs.

## Introduction

Extensive rostral mandibular defects can be secondary to trauma, tumor resection, or other pathologic, developmental, or congenital disorders. Rostral mandibular critical-size bone defects (i.e., an osseous defect that would not heal by bone formation during the lifetime of the animal because of the extent of the defect) result in malocclusion and instability (1–7). Extensive loss of bone at this region can result in difficulty with prehension and mastication. Furthermore, loss of rostral mandibular continuity due to resection and subsequent malocclusion may influence the mandibular head–mandibular fossa congruity and result in pain and degeneration of the temporomandibular joint (TMJ) (1–3, 8–10). Importantly, reconstruction of the rostral mandible can be challenging due to the complex anatomical geometry of this region. Particularly, the shape of the rostral mandibles in dogs resembles a sharp-angled arc, which is quite different from the geometric shape of the mandibular body and the rounded conformation in humans (11).

Mandibular reconstruction of critical-size defects requires rigid fixation, typically in the form of a plate and screws, and well-vascularized soft tissues. There are several strategies to fill the critical-size bone defects including autologous bone grafts, bone graft substitutes, and free-fibular flap tissue transfer (4, 11–14). However, these methods are not ideal as they result in donor site morbidity, are limited by graft size (especially in small dogs), and are difficult to contour (12, 15, 16). In addition, the outcome of the aforementioned may be unpredictable. Our group and others have demonstrated that a regenerative approach to reconstruction of mandibular critical-size defects in dogs using a scaffold and growth factors such as rhBMP-2 can be performed successfully and represents an excellent functional solution (3, 17–19). Furthermore, regenerating the mandibular bone allows restoration of continuity and, therefore, proper biomechanics and functional pain-free occlusion (12, 20).

Bone regeneration using bioactive compounds and a bioengineered scaffold has been studied extensively with variable rates of success. The landmark study was pioneered by Urist over 40-years-ago where he discovered that bone morphogenetic proteins (BMPs) are bioactive compounds responsible for bone regeneration (21, 22). This work demonstrated that it is feasible to harness the native regenerative capacity of the body with exogenous signals to generate autologous tissue of pre-specified shape (23). Later, Reddi further demonstrated that BMPs are responsible for the signaling cascade of events that lead to induction of progenitor cells into new bone formation (24, 25). These exciting discoveries have led to the clinical use of BMPs in the fields of fracture healing, engineering of dental tissues, and spinal fusion (26, 27). Furthermore, at present date, rhBMP-2 or rhBMP-7 delivered by implantable collagen matrices is FDA approved for spinal fusion (26, 28–30).

We previously described reconstruction of segmental mandibular bone defects that resulted from non-union fractures or tumor resections using titanium locking plates and rhBMP-2 delivered in a compression resistant matrix (CRM) scaffold in dogs (17, 19). Therefore, we have extended this surgical approach to reconstruction of the rostral mandibles following extensive rostral mandibulectomy. Here, we describe a case series of five dogs that underwent bilateral rostral mandibular reconstruction following mandibulectomy using internal fixation and a CRM impregnated with rhBMP-2. In addition, we report the important use of 3D biomodel printing as a surgical planning tool.

## Materials and Methods

### Case inclusion

Dogs requiring extensive rostral mandibulectomy due to odontogenic or non-odontogenic tumors that were presented to William R. Prichard Veterinary Medical Teaching Hospital, University of California Davis, Davis were included in this case series report. Informed consent was obtained from the dog’s owners. The pre-surgical workup for all dogs included minimal data base (i.e., complete blood count, serum biochemistry, and urinalysis) and staging by means of abdominal ultrasound, and thoracic radiography or computed tomography (CT) (31). Furthermore, the lymph nodes were evaluated by contrast CT and fine-needle aspiration for cytological analysis. Postoperatively, the dogs were evaluated at regular intervals for the duration of the reported follow-up period.

### Computed tomography and 3D model printing

Transverse, 0.625-mm, collimated CT images of the heads, with and without contrast, were obtained for all dogs before surgery and immediately postoperatively. For two dogs, an additional CT was performed 6 months postoperatively and for one dog follow-up CT was performed 2 months postoperatively. The CT was performed using a LightSpeed 16 (GE Healthcare, Milwaukee, WI, USA) CT scanner with kVp = 120 and auto-mA. All images were reconstructed using a bone filter. A CT calibration phantom containing five reference rods of known density (Mindworks Software, Inc.; San Francisco, CA, USA) was included in the field of view during image acquisition.

Computed tomography images were evaluated qualitatively and quantitatively using DICOM viewing software (OsiriX v. 4.1.2 32-bit; Geneva, Switzerland) and data analysis software (MATLAB R2013a; Mathworks^®^, Natick, MA, USA). The volume of mineral repair tissue, average mineral density, and porosity were measured for the rostral mandibular repair immediately postoperatively and at 6 months. Following image calibration, the volume of mineral repair tissue was calculated by determining the number of pixels with mineral density values between 255 and 1260 mg K_2_HPO_4_/mL in a region of interest (ROI) drawn to include the CRM scaffold, associated surgical implants, newly formed bone, and adjacent soft tissues. Values in this mineral density range excluded pixels representing soft tissue or metal. The number of mineral pixels was counted for all transverse CT images rostral to the mandibulectomy sites; the volume of mineral repair tissue was then calculated by multiplying the number of mineral pixels by the individual voxel volume. Average mineral density and porosity of the native bone and repair tissue were determined from four representative, non-consecutive, transverse CT images using freeform ROIs that included the native bone or CRM scaffold/repair tissue, but excluded teeth and metal surgical implants. Measurements from the four images were averaged to reduce error associated with measurement and image-to-image variability. Porosity was calculated as the number of pixels with mineral density <255 mg K_2_HPO_4_/mL divided by the total number of pixels in the ROI.

**Figure 1:**
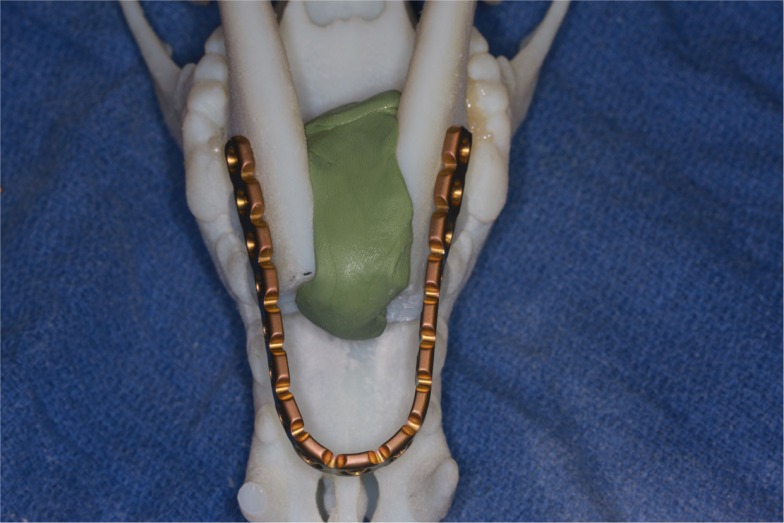
**Surgical planning on a 3D printed skull demonstrating the adjustment and adaptation of the titanium locking plate to the model of the rostral mandibles**.

For all patients, 3D volume renderings of the transverse CT images were generated for surgical planning. Next, a surface rendering of the bones was created from the transverse DICOM images and compiled into a Standard Tessellation Language (STL) mesh. A 3D model was then printed at exact scale using an Objet Connex 260V Polyjet Printer (Objet/Stratasys, Rehovot, Israel). The surgical procedure was performed on the 3D model prior to surgery in three dogs and a single titanium locking plate (2.4/3.0 mm, Synthes^®^ Maxillofacial, Paoli, PA, USA) was contoured in a horseshoe shape to extend rostrally to the level of the maxillary first premolar–canine teeth (Figure 1). Based on our understanding of the skull configuration of dogs, we decided that bending the plate in a sharp angle may lead to early plate breakage and should be avoided. In two cases, the plate was contoured without a 3D model during the surgery.

### CRM and rhBMP-2 preparation

The CRM and rhBMP-2 were prepared as previously described (17, 19). Briefly, CRM [collagen sponge with embedded granules of hydroxyapatite (HA) and tricalcium phosphate, MasterGraft Matrix^®^ Medtronic, Memphis, TN, USA] and rhBMP-2 (Medtronic, Memphis, TN, USA) were used in this study. The volume of the defect was measured in three dimensions and a sufficient amount of CRM to provide half to three-quarters of the mandibular height and a length 2 mm greater than the defect span was measured. Ten minutes prior to implantation, the CRM was infiltrated with a 0.5 mg/mL solution of rhBMP-2 at a volume corresponding to 50% of the volume of the prepared CRM scaffold. For example, for a scaffold that was 4.5 cm in length, 1 cm mandibular width, and 1.5 cm mandibular height (4.5 cm × 1 cm × 1.5 cm), the total defect volume was 6.75 cm^3^; thus, 3.38 mL of the rhBMP-2 solution was used.

### Surgical technique

**Figure 2:**
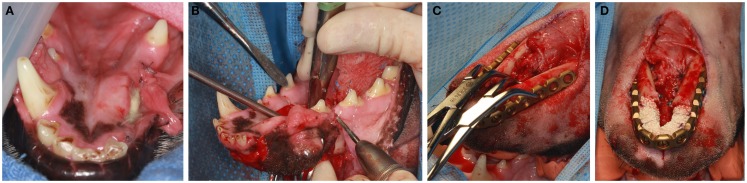
**With the dog in sternal recumbency intraoral approach (A,B) is performed for the osteotomy**. Once osteotomy is completed, the dog is positioned to a dorsal recumbancy and extraoral approach **(C,D)** is used for plate adaptation **(C)**. Once the plate is secured with titanium locking screws, the CRM infused with rhBMP2 is implanted in the defect and secured to the plate **(D)**.

Prior to surgery pharyngotomy intubation was performed as previously described (32). Then, a bite registration of the caudal dentition, from distal to the mandibular fourth premolar teeth up to the third mandibular molar teeth was obtained using vinyl polysiloxane impression material putty (3M ESPE, St. Paul, MN, USA). This was performed to precisely capture the dental occlusion before the bilateral rostral mandibulectomy. The mandibular area was clipped and surgically prepared for aseptic surgery. Ampicillin 20 mg/kg was administered IV preoperatively. Bilateral rostral mandibulectomy was performed with the dog in sternal recumbency as previously described (Figure 2A) (32). The resection area was measured and marked with a surgical marking pen. Then, the rostral mandibles, including bone and soft tissues, were resected (Figure 2B) ensuring appropriate surgical margins (10 mm or more) followed by an intraoral closure in a single layer using 4–0 poliglecaprone 25 (Monocryl^®^, Ethicon, Somerville, NJ, USA). The dogs were then placed into dorsal recumbency and the previously obtained impression placed in the mouth to recapture the normal occlusion of the remaining mandibles. An extraoral approach to both mandibles was made via a single midline incision. Following sharp and blunt dissection, the mandibles were exposed and the previously contoured plate (2.4/3.0 mm mandibular locking reconstruction plate, Synthes^®^ Maxillofacial, Paoli, PA, USA) was adjusted and adapted to the bone with bone forceps (Figure 2C) and then secured with 3-mm locking screws. The planning of plating should take into consideration the shortage of skin following amputation and that the skin should not be stretched over the plate. Importantly, in order to avoid iatrogenic teeth trauma, the plate was positioned ventral to the roots of the teeth. Prior to implantation of the rhBMP-2 infused CRM, the surgical site was copiously irrigated with sterile saline. The infused CRM was then implanted in the defect to fit snugly and secured circumferentially with 4–0 poliglecaprone 25 (Monocryl^®^, Ethicon, Somerville, NJ, USA) to prevent migration post-implantation (Figure 2D). The surrounding soft tissues were sutured around the plate and CRM to provide a soft tissue envelope. The subcutaneous tissues and skin were closed routinely.

Postoperative care included soft food for 2 weeks and administration of amoxycyllin/clavulanic acid 20 mg/kg orally (Clavamox, Pfizer Animal Health, NY, USA) twice daily for 2 weeks. Analgesia was achieved by administration of opioids and non-steroidal anti-inflammatory medications for 7–14 days.

## Results

Summary data for the dogs are provided in Table 1. Overall, five dogs aged 3–10 years (mean 6.8 years) weighting 22.2–64.5 kg (mean 34.9 kg) that received rostral mandibulectomy and reconstruction were included. All dogs were in good physical condition. Results of hematological, serum biochemical analysis, and urinalysis were generally considered normal for other then one dog had preexisting and well-managed stage 3 chronic kidney disease. Thoracic CT and abdominal ultrasonography performed during tumor staging revealed no abnormalities in all dogs. No intraoperative complications occurred in any dog and the surgical margins were confirmed to be free of neoplastic cells by histopathological analysis. For one dog with squamous cell carcinoma, a staged procedure was performed due to the size and extent of the tumor. The first stage consisted of a bilateral rostral mandibulectomy with wide (minimum of 10 mm) resection of skin and oral mucosa. Once the histopathology results confirmed tumor-free margins, a second surgery was performed 4 weeks later to reconstruct the rostral mandibles as described above.

**Table 1:** **Summary data for five dogs that received rostral mandibular reconstruction**.

Dog	Age (years)	Weight (kg)	Breed	Tumor type	Follow-up (months)
1	8	38.2	Great Dane mix	Ossifying fibroma	24
2	10	25.7	Collie	Squamous cell carcinoma	10
3	4	23.7	Labrador retriever	Squamous cell carcinoma	2
4	9	22.2	Standard poodle	Peripheral odontogenic fibroma	2
5	3	64.5	Neapolitan mastiff	Acanthomatous ameloblastoma	9

### Follow-up

**Figure 3:**
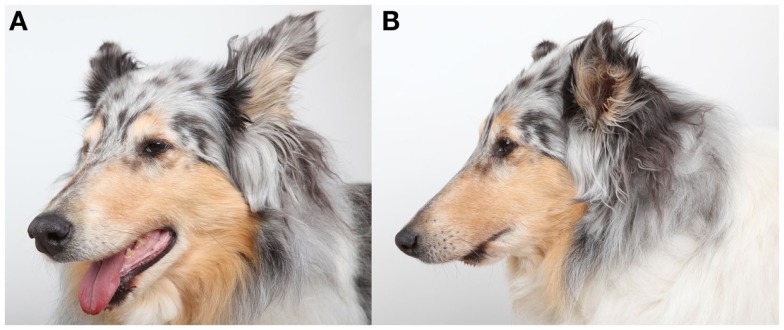
**Photographs of dog 2 at 6 months recheck illustrating the appearance of the mandibles and skull following successful reconstruction of the rostral mandibles**.

The follow-up period was 2–24 months (mean 9.4 months). All dogs were examined clinically by means of oral examination and palpation immediately postoperatively and throughout the duration of the follow-up period and were found to have appropriate occlusion (Figure 3). Furthermore, all dogs had immediate return to normal activity apart from restriction of heavy chewing for 2–3 months. Two weeks postoperatively, hard tissue spanning the entire defect site was palpable and covered by intact gingiva in three dogs. At 4 weeks postoperatively, the defect felt completely solid and no abnormalities were noticed. At the reported follow-up periods, palpation of the reconstruction area revealed the presence of hard tissue with no recurrence of the tumors.

### Complications

One dog exhibited plate exposure through the mucosa 14 days following surgery. A mucosal flap adjacent to the plate exposure was prepared and following copious irrigation with sterile saline, the plate was covered and the flap sutured using 4–0 poliglecaprone 25 (Monocryl^®^, Ethicon, Somerville, NJ, USA) in a single interrupted fashion. Some of the scaffold material in the proximity of the rostral part of the plate was removed during irrigation. One dog exhibited wound dehiscence 6 days postoperatively and the CRM material was dislodged. A revision surgery was performed with resuturing of the skin and the oral mucosa. Due to the presence of contamination and the possibility of infection, implantation of a new rhBMP-2-infused CRM was not performed. Eight weeks later, an oral examination under general anesthesia was performed and CT and dental radiographs were obtained. Clinically, appropriate occlusion, intact mucosal covering were observed and hard tissue formation was palpated at the rostral mandibles. CT revealed an intact bone plate and bone screws with no evidence of osteolysis of the native mandibles. The CRM scaffold was no longer present, but had been replaced by homogeneous, smoothly margined, mineral opacity tissue bridging the intermandibular space between the right and left mandibular ostectomy sites. This mineral opacity tissue was distinct in appearance from the original CRM scaffold, contiguous with the native mandibles, and consistent with regenerating bone. The mineral volume of the original CRM scaffold measured 2.65 cm^3^ with an average mineral density of 460.8 mg K_2_HPO_4_/mL and porosity of 8.8%. Despite complete loss of the CRM scaffold, the newly formed bone 8 weeks after revision surgery had a mineral volume of 1.87 cm^3^, average mineral density of 490.4 mg K_2_HPO_4_/mL, and porosity of 6.5%. A small (~6–9 mm) gap persisted between the rostral extent of the new mineral opacity tissue and the rostral aspect of the bone plate. During the same time interval, native mandible increased in average mineral density from 769.2 to 844.5 mg K_2_HPO_4_/mL and decreased in porosity from 22.4 to 18.6%. Overall, recheck CT findings demonstrated healing of the mandibulectomy sites without any evidence of osteomyelitis associated with the post-surgical wound dehiscence.

For the remaining follow-up period, no other abnormalities were noticed and no plate exposure through the mucosa was noted. Furthermore, all owners reported that the dogs had an excellent quality of life.

### CT evaluation

**Figure 4:**
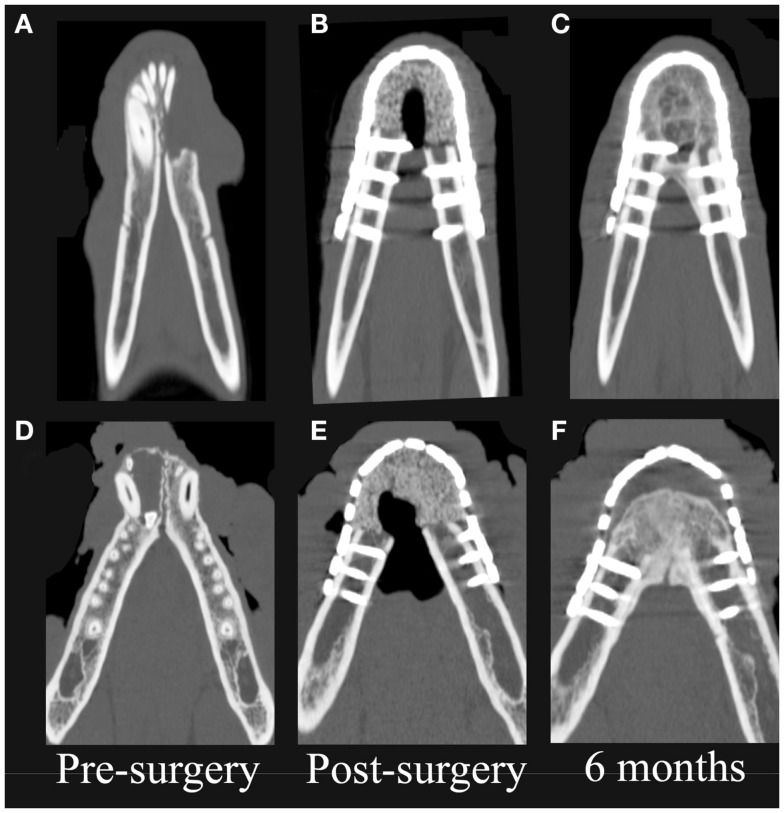
**Dorsal-plane, reconstructed, CT images of the mandibles in two patients prior to surgery (A,D), immediately postoperatively (B,E), and 6 months after surgery (C,F)**. The right side of the patient is displayed at the left side of each image. Note geographic osteolysis of the rostral left mandibles in patient 1 **(A)** and of the rostral right mandibles in patient 2 **(D)** associated with squamous cell carcinoma and acanthomatous ameloblastoma, respectively. After 6 months, the borders between the scaffold and the native mandibles have become less distinct and new regenerated osseous tissue fills the rostral intermandibular space.

**Figure 5:**
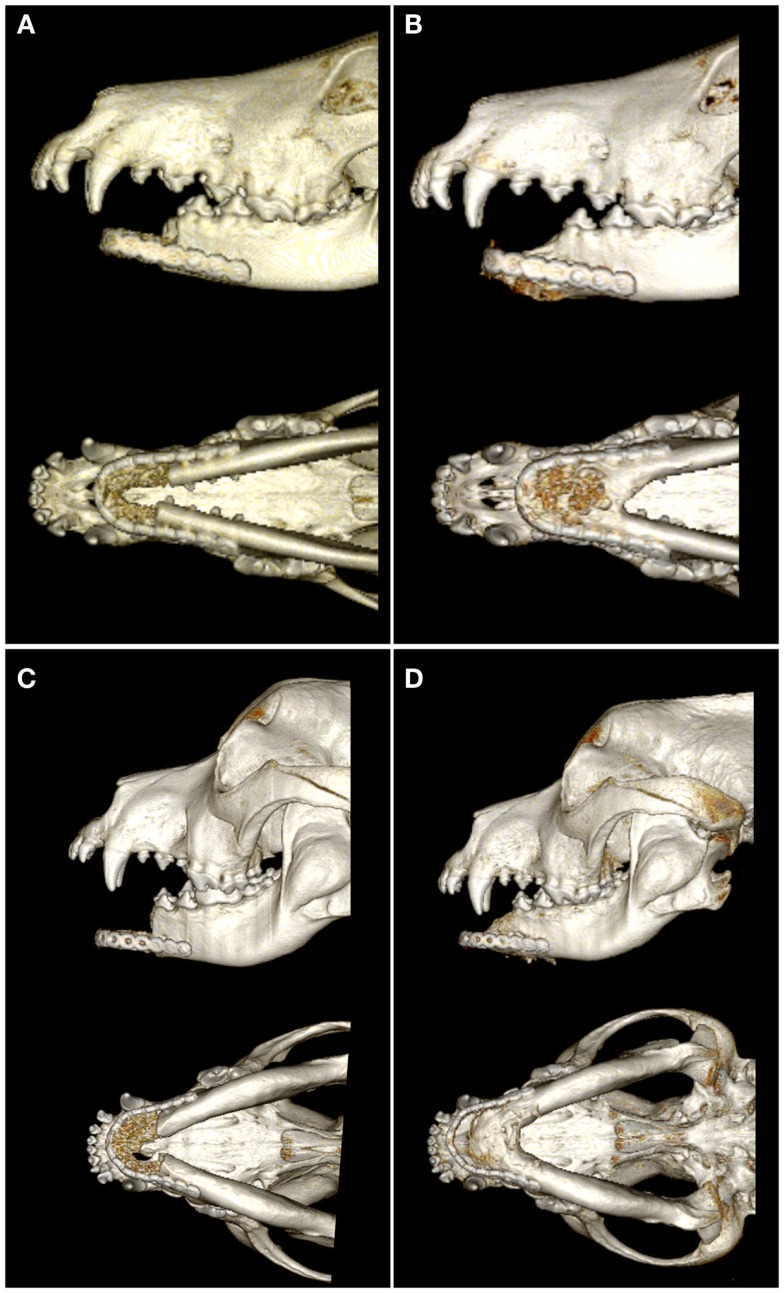
**Lateral and ventral views of 3D volume renderings created from the CT images immediately postoperatively (A,C) and 6 months after surgery (B,D)**. Note the new bone formation in the intermandibular space and bridging the ostectomy sites of both patients 6 months after surgery.

On CT images, there was radiologic evidence of smooth to mildly irregular new bone formation at the axial, ventral, and dorsal surfaces of the implant material (Figures 4 and 5). The ostectomy sites were much less distinct at 6 months compared to immediate postoperative CT. Furthermore, new bone bridged between the axial and ventral aspects of the native mandible and the implant material at 6 months, consistent with integration between the implant material and native mandible (Figures 4C,F). One dog demonstrated a large amount of heterogeneous, smoothly margined, hypoattenuating mineral opacity repair tissue filling the rostral intermandibular space 6 months after surgery (Figure 4C). In the one dog requiring removal of a portion of the implant material, a small gap was visible on CT images between the rostral aspect of the curved bone plate and the implant material (Figure 4F).

Quantitative CT measurements in the two dogs that did not exhibit complications indicate remodeling of both the native mandibular bone and the CRM scaffold/repair tissue. Importantly, both patients exhibited an increase of 43–53% in the volume of mineral repair tissue between postoperative CT and the 6-month recheck CT. The average mineral density (483 ± 16–518 ± 52 mg K_2_HPO_4_/mL) and porosity (7.4 ± 2.3–9.3 ± 5.9%) of the CRM scaffold were similar in both patients immediately after surgery. The scaffolds were less dense and less porous compared to native mandible, which had average mineral density of 577 ± 5–713 ± 47 mg K_2_HPO_4_/mL and porosity of 27.0 ± 4.0–33.0 ± 2.0%. Both patients exhibited decreased average mineral density (−11.0 to −23.0%) and increasing porosity (+6.0 to +10.0%) of the repaired tissue at the 6-month CT. Native mandible had essentially unchanged mineral density (+2.0%) and a 6.6% reduction in porosity in one patient during the recheck interim while the native mandible decreased in average mineral density by 16.0% and increased in porosity by 10.4% in the second patient. Despite differences in the response of the native mandible in the two patients, the repair tissue demonstrated increased volume of mineral, decreased average density, and increased porosity in both patients.

## Discussion

This is the first report on a series of dogs that underwent immediate or delayed bilateral rostral mandibular reconstruction using internal fixation and a CRM infused with rhBMP-2. Furthermore, we report our surgical technique and clinical experience on the use of rhBMP-2 in bilateral rostral mandibular reconstruction as well as reporting the clinical and radiological outcome. Importantly, this report exemplifies the benefits of a regenerative approach to reconstruction of mandibular bone defects in dogs.

Extensive bilateral rostral mandibulectomy results in mandibular brachygnathism and instability of the remaining mandibles (32). In addition, since the rostral support to the tongue is resected, the tongue protrudes from the mouth and drooling occurs. Overall, the more extensive the resection, the more pronounced are the functional complications. Significantly, the presence of mandibular instability results in abnormal mechanical stress applied to the TMJ, which in turn may cause degeneration of the joint (10, 33, 34). While degenerative changes to the joint may not be clinically noticeable in the immediate period following surgery, long-term TMJ degeneration can cause pain and dysfunction (35, 36). Therefore, the ideal solution to functional complications following extensive bilateral rostral mandibulectomy is reconstruction and restoration of continuity as much as possible.

Reconstruction of segmental mandibular bone defects due to tumor resection or following defect non-union fractures are performed at our institute using a regenerative approach (17, 19). We previously reported that this combined surgical and regenerative strategy resulted in a rapid return to normal function. Although there have been attempts to eliminate mandibular instability following rostral mandibulectomy via the use of orthopedic pins, screws, and bone grafts, none of these approaches resulted in reconstruction of the continuity of the rostral mandibles (32, 37, 38). Therefore, adopting a regenerative surgical approach to reconstruction of the normal, or near normal, mandibular anatomy, and occlusion is crucial to reestablishing the proper mandibular biomechanics and pain-free functionality. However, as seen in this report, reconstruction of the full length of the mandible may not be achievable due to shortage of soft tissue following amputation and bending the plate in a sharp angle may lead to early implant failure or breakage of the plate.

In agreement with our previous reports, solid tissue formation was palpable as early as 2 weeks following surgery. By 6 months, this tissue appeared radiographically well-integrated, but was less dense and less porous than native mandible. Although we did not examine the histological characteristics of this new bone formation, previous reports confirmed that rhBMP-2 infused on a CRM scaffold resulted in mineralized trabecular bone development reflective of healthy bone turn over and remodeling (3, 39, 40). CT examination at more frequent and later time points following surgery would be necessary to determine if and at what rate the regenerated mandibular tissue achieves the density and porosity of native bone.

Regeneration of critical-sized bone defects requires an ability to recapitulate developmental biology processes and control tissue morphogenesis (41). In addition, the development of functional bone through a regenerative approach depends on the delivery of physical and chemical cues (41). These cues were delivered by rhBMP-2 imbedded in a CRM scaffold. Furthermore, rhBMP-2 is responsible for replicating the native microenvironmental cues in a spatiotemporal manner to provide adequate localized osteoinduction. However, the application of rhBMP-2 critically depends on the scaffold and the dosage concentration and time of application (42, 43). The scaffold used in this study is a CRM that was used successfully in several other reports (3, 17–20, 30). Noteworthy is that the dose used in this study (0.5 mg/mL with a 50% soak volume) is the same dose used in previous reports and, therefore, is our recommended clinically appropriate dose in dogs (3, 17, 19). However, higher dosage of rhBMP-2 may result in excessive bone formation (44). Nevertheless, long-term follow-up on the use of rhBMP-2 for rostral mandibular reconstruction in dogs is required to understand the bone remodeling, bone regeneration, and its possible affect on the surrounding soft tissues.

An unfortunate complication was observed in two dogs with partial plate exposure through the oral mucosa and wound dehiscence. It is possible that the rostral location of the plate and/or the lack of robust connective tissue (i.e., muscles, fat, thick submucosa) to cover the plate may be the reasons for these complications. Plate exposure through the mucosa was first described under experimental condition (45). Furthermore, different reports also described plate exposure through the mucosa in dogs that had segmental reconstruction surgery. However, in these reports the dogs received two plates to buttress the defect (3, 8). In these cases, the plate exposure was resolved by plate removal (3, 8). In the present series of dogs, we demonstrated that, if exposed through the mucosa, a flap procedure in concert with copious irrigation with sterile saline may salvage the titanium locking plate. One of the dogs in this report had a staged procedure with rostral mandibulectomy performed 4 weeks prior to reconstruction due to the size and extent of the tumor and the attempt to verify tumor-free surgical margins by histopathology. It is prudent to stress that placement of a growth factor such as rhBMP-2 in a surgical site with persistent tumor cells is contraindicated and is likely to contribute to rapid recurrence of the tumor. Therefore, we recommend that careful patient selection be considered based on the size, biological behavior, and invasive nature of the tumor.

The present study exemplifies the use of 3D printing as a surgical planning modality for mandibular reconstruction in dogs. Reconstruction of the maxillofacial region can be challenging even to the experienced surgeon due to its complex geometry (46). We found that having a 3D model provided the surgeon with the ability to perform precise preoperative planning and practice a virtual osteotomy and design a patient-specific implant preoperatively (46–48). While radiological 3D visualization is pivotal for the discipline of maxillofacial reconstruction, it is limited to the use of a flat screen. 3D printing of the affected skull overcomes this limitation and allows for a tangible understanding of the disorder and the precise surgical treatment (46). This may be further justified as precise pre-surgical planning may reduce the surgery time and allow for a reduction in overall surgical costs (49).

In conclusion, a regenerative approach to rostral mandibular reconstruction, as was demonstrated here using a CRM infused with rhBMP-2 is possible and with predictable good outcome. Furthermore, incorporating 3D printing as part of the surgical planning is important and beneficial for graspable understanding of the disorder and for precise surgical treatment. Based on the present and previous reports, the realm of regenerative surgical reconstruction of mandibular critical-size defects in dogs is justifiable as it provides reproducible and predictable new bone growth and avoids the need for harvesting autologous bone and associated morbidity and pain.

## Conflict of Interest Statement

The authors declare that the research was conducted in the absence of any commercial or financial relationships that could be construed as a potential conflict of interest.
